# IUP-BERT: Identification of Umami Peptides Based on BERT Features

**DOI:** 10.3390/foods11223742

**Published:** 2022-11-21

**Authors:** Liangzhen Jiang, Jici Jiang, Xiao Wang, Yin Zhang, Bowen Zheng, Shuqi Liu, Yiting Zhang, Changying Liu, Yan Wan, Dabing Xiang, Zhibin Lv

**Affiliations:** 1 College of Food and Biological Engineering, Chengdu University, Chengdu 610106, China; 2 Department of Medical Instruments and Information, College of Biomedical Engineering, Sichuan University, Chengdu 610041, China; 3 Country Key Laboratory of Coarse Cereal Processing, Ministry of Agriculture and Rural Affairs, Chengdu 610106, China; 4 College of Biology, Southwest Jiaotong University, Chengdu 610031, China; 5 College of Biology, Georgia State University, Atlanta, GA 30302-3965, USA

**Keywords:** umami peptide, prediction, deep learning, BERT, SMOTE

## Abstract

Umami is an important widely-used taste component of food seasoning. Umami peptides are specific structural peptides endowing foods with a favorable umami taste. Laboratory approaches used to identify umami peptides are time-consuming and labor-intensive, which are not feasible for rapid screening. Here, we developed a novel peptide sequence-based umami peptide predictor, namely iUP-BERT, which was based on the deep learning pretrained neural network feature extraction method. After optimization, a single deep representation learning feature encoding method (BERT: bidirectional encoder representations from transformer) in conjugation with the synthetic minority over-sampling technique (SMOTE) and support vector machine (SVM) methods was adopted for model creation to generate predicted probabilistic scores of potential umami peptides. Further extensive empirical experiments on cross-validation and an independent test showed that iUP-BERT outperformed the existing methods with improvements, highlighting its effectiveness and robustness. Finally, an open-access iUP-BERT web server was built. To our knowledge, this is the first efficient sequence-based umami predictor created based on a single deep-learning pretrained neural network feature extraction method. By predicting umami peptides, iUP-BERT can help in further research to improve the palatability of dietary supplements in the future.

## 1. Introduction

Umami taste determines the deliciousness of foods. Many foods possess umami ingredients, such as meat products [1,2], mushroom [3], soy sauce [4], seafoods [5], and fermented foods [6]. In addition to sweet, bitter, salty, and sour, umami taste was recognized as the fifth taste, which is characterized as a meaty, savory, or broth-like flavor [7]. The perception of sweet, bitter and umami taste is inspired by the binding of taste components to the G protein-coupled receptor [8,9]. The main umami taste receptor is an independent heterodimeric T1R1/T1R3 receptor [10,11]. Umami ingredients are widely used in food production, with several health benefits [12]. Umami peptides are a group of specific structural peptides, which endow foods with a favorable umami taste [6]. The primary structure of umami peptides is usually short linear peptides, with a molecular weight distribution of less than 5000 Da. Dipeptides and tripeptides account for approximately 60% of the isolated umami peptides [3,10]. Longer linear peptides, including pentapeptides, hexapeptides, heptapeptides, and octapeptides, were also discovered to possess strong umami intensity [1,2,5,13]. The binding mechanism of umami peptides to the taste receptor was distinguished from that of other umami ingredients, indicating their special taste attributes [10,14]. Moreover, umami peptides displayed synergy with typical umami substances, such as monosodium glutamate (MSG) [15]. Some showed umami-enhancing effects in MSG or NaCl solution [16]. Several health benefits, including reducing dietary salt content, antioxidant activity [17], inhibiting the activities of dipeptidyl peptidase-IV [18] and angiotensin I converting enzyme [17,18], have been reported for umami peptides. Therefore, umami peptides could be a good supplement to other traditional umami substances and display prospective application in the food seasoning industry. 

The existing laboratory approaches used to identify and characterize umami peptides, including RP-HPLC [19], MALDI-TOF-MS [13], LC-Q-TOF-MS [3], and UPLC-ESI-QTOF-MS/MS [20] analyses, are time-consuming and labor-intensive, which restrict the high-throughput and rapid screening of umami peptides. Therefore, applying accurate and efficient computer-assisted methods to identify umami peptides is a necessity and complementary to the experimental methods [21]. The knowledge of the interaction of umami peptides with the taste receptors promoted the search for new novel umami peptides. Computational approaches, such as molecular docking and homology modeling, have been applied to identify umami peptides [21,22]. By conjugating estimated propensity scores of amino acids and dipeptides with the scoring card method (SCM), the first sequence-based umami peptide predictor, namely iUmami-SCM (http://camt.pythonanywhere.com/iUmami-SCM (accessed on 14 November 2020)) [23], was developed. It analyzes and predicts umami sensory peptides merely based on the information of the primary peptide sequence, without knowing the advanced structure. IUmami-SCM afforded sensitivity (Sn), the deduced balanced accuracy (BACC), and Matthew’s coefficient correlation (MCC) of 0.714, 0.824, and 0.679, respectively. Nevertheless, the artificial feature extraction method and only a single type of feature was used as the input of machine learning (ML) models. Consequently, the sequence feature information of iUmami-SCM is insufficient and the performance is not very satisfactory. Recently, the ML-based umami peptide meta-predictor, namely UMPred-FRL [24], was created based on a feature representation learning approach, with an open-access web server at http://pmlabstack.pythonanywhere.com/UMPred-FRL (accessed on 20 December 2021) Seven different feature encodings, comprising amino acid composition, dipeptide composition, composition transition-distribution, amphiphilic pseudo-amino acid composition, and pseudo-amino acid composition, were conjugated with the six well-known ML algorithms (k-nearest neighbor (KNN) [25], extremely randomized trees, partial least squares, random forest (RF) [26], logistic regression (LR) [27], and support vector machine (SVM) [28,29]) Compared with its baseline models, higher accuracy was achieved on the benchmark dataset. It also outperformed the iUmami-SCM method consistently on the independent test dataset [24]. Yet, the overall prediction performance of UMPred-FRL is still not efficient enough, with accuracy (ACC) to be 0.888, MCC to be 0.735, Sn to be 0.786, and BACC to be 0.860. This may be caused by an inefficient manual ML feature exaction method being used. Therefore, for rapid and specific umami peptide screening, more robust, accurate, and higher sensitivity prediction models are needed.

Deep learning is an algorithm in ML, which enables the computer to learn to use features while learning how to extract features: Learn how to learn [30]. It could automatically transform raw protein sequences into a form utilized effectively by ML, without the need of preprocessing or prior characterization of data. It is now increasingly being adapted in protein recognition, where complex informatics pipelines could be replaced with models that predict structures directly from sequences [30]. Bidirectional encoder representations from transformer (BERT) refers to a transformer-based deep learning method created by Google for pretraining natural language processing [31,32]. The core of BERT is a transformer language model with a variable number of encoder layers and self-attention heads. It takes use of a new masked language model and can generate deep bidirectional language representations, providing a pretraining and fine-tuning approach, using enormous amounts of unlabeled data. BERT creates general-purpose understandings first before using task-specific data to address a variety of applications with the least amount of architectural change. After pretraining, an additional output layer was added for fine-tuning, and a state-of-the-art performance was obtained for various downstream tasks [32]. With a global receptive field, BERT can effectively capture more global context information than the convolutional neural network-based models. Recently, BERT has achieved gratifying results in the prediction of various functional peptides, such as bitter peptides [33], antimicrobial peptides [34], and human leukocyte antigen peptides [35]. Soft symmetric alignment (SSA) has defined a brand-new method to compare arbitrary-length sequences within vectors [36]. An initial pretrained language model is used to encode a peptide sequence, as a three-tier stacked BiLSTM encoder output is meanwhile utilized. Each peptide sequence creates the final embedding matrix by employing a linear layer, $R^{L\times 121}$, in which L represents the peptide length. In the SSA embedded model, the model was trained and optimized using the SSA strategy [37,38].

Here, we created a novel ML-based predictor, namely iUP-BERT, which employed a deep learning pretrained neural network feature extraction method for model development. For model performance improvement, the synthetic minority oversampling technique (SMOTE) [39] was applied first to overcome the data imbalance. To achieve higher prediction accuracy, the pretrained sequence embedding technique SSA or BERT was then combined with five different ML algorithms (KNN, LR, SVM, RF, and light gradient boosting machine (LGBM) [38]) to build several models. The features of the BERT method combined with the SVM model were finally selected and used to raise the prediction efficacy after optimization. The results from both the 10-fold cross-validation and independent test represented that the application of the deep representation learning BERT method remarkably improved the model performance in identifying umami peptides. IUP-BERT achieved higher accuracy than existing methods based on peptide sequence information alone.

## 2. Materials and Methods

### 2.1. Overall Framework

**Figure 1:**
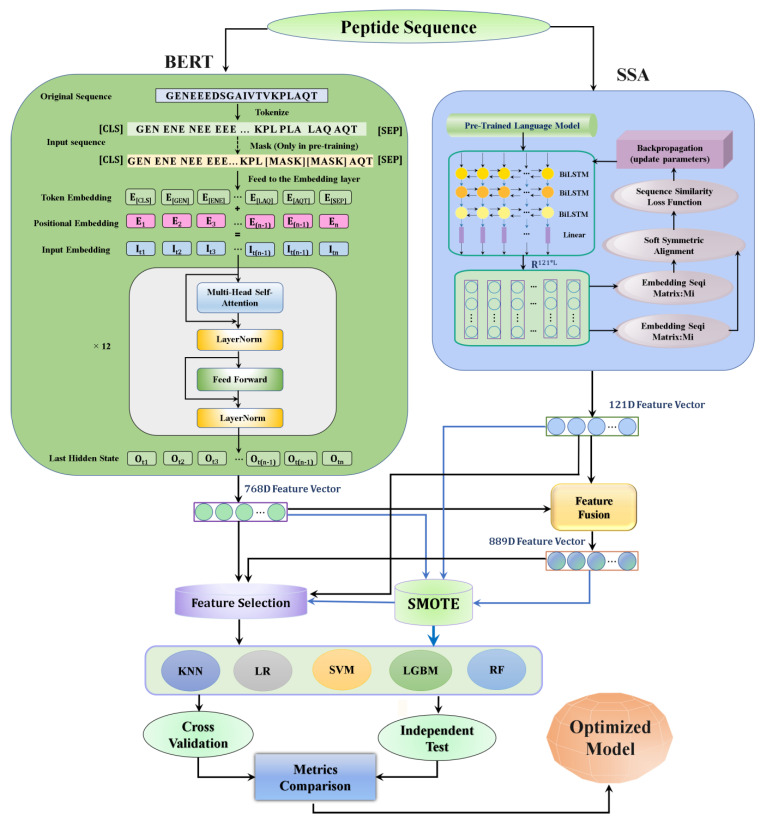
Overview of iUP-BERT development. The illustration depicts the 6 main steps for model development. (1) The peptide sequence was included as text and feature-extracted by the BERT model and SSA method. (2) The 788D BERT extracted feature was fused with the 121D SSA extracted features to make an 889D fusion feature vector, with individual feature vectors as comparison. (3) The SMOTE method was used to overcome the data imbalance. (4) The LGBM feature selection method was used to attain the best feature combinations. (5) Five different ML algorithms (KNN, LR, SVM, RF, and LGBM) were combined with the above techniques to build several models. (6) The final iUP-BERT predictor was established by combining the optimized feature representations. Here, BERT is for Bidirectional Encoder Representations from Transformers; SSA is for Soft Sequence Alignment; SMOTE: Synthetic Minority Oversampling Technique; LGBM is for Lighting Gradient Boosting Machine; D is for Dimension; KNN is for K-Nearest Neighbors; LR is for Logistic Regression; SVM is for Support Vector Machine; RF is for Random Forest.

Figure 1 illustrates the overall framework of iUP-BERT. The main steps are as follows:

1. Upon the introduction of the peptide sequence, the pretrained sequence embedding technique, BERT, was used for feature extraction. For comparison, the SSA sequence embedding technique was included.
2. After the feature extraction, BERT was fused with SSA to make an 889D fusion feature vector.
3. The SMOTE was used to overcome the data imbalance.
4. For feature space optimization, the LGBM feature technique method was used.
5. Five different ML algorithms (KNN, LR, SVM, RF, and LGBM) were combined with the above techniques to build several models. The features of the BERT-SMOTE-SVM model were selected and applied to raise the prediction accuracy after optimization.
6. The optimized feature representations were combined to establish the final iUP-BERT predictor.

### 2.2. Datasets

For fair comparison, the same peptide datasets (Supplementary File S1) used in previous umami peptide ML models were chosen [24]. In the datasets, 140 peptides either from experimentally validated umami peptides [10,15,16,20] or from BIOPEP-UWM databases [40] were taken as positive samples, whereas the negative samples were 302 non-umami peptides, identified as bitter peptides [41,42]. All peptide sequences in both the positive and negative samples were unique. The training dataset includes 112 umami and 241 non-umami peptides. The independent test dataset contains 28 umami and 61 non-umami peptides. 

### 2.3. Feature Extraction

To extract different and effective features on umami peptide recognition, two deep representation learning feature extraction methods, the pretrained SSA sequence embedding model and the pretrained BERT sequence embedding model, were used. Meanwhile, the dataset was either pretrained with the SMOTE embedding model or not. To identify specific umami peptides, the models were trained on an alternate dataset. More comprehensive predictive models were created after comparison of different feature encoding schemes.

#### 2.3.1. Pretrained SSA Embedding Model

SSA defines a brand-new approach to compare arbitrary-length sequences within vectors [36]. An initial pretrained model is utilized to encode a peptide sequence, as a three-tier stacked BiLSTM encoder output is utilized meanwhile (Figure 1) Each peptide sequence creates the final embedding matrix by employing a linear layer, $R^{L\times 121}$, in which L represents the peptide length. A model like this, which was trained and optimized by the SSA method, is called an SSA embedded model.

Consider two embedded metrics of $R^{L\times 121}$, with the names $P_{1}$ and $P_{2}$ for two distinct peptide sequences with varying lengths, $L_{1}\;$ and ${\;L}_{2}$

(1) $$P_{1}=\left[\alpha_{1},\alpha_{2},\cdots ,\alpha_{L1}\right]$$

(2) $$P_{2}=\left[\beta_{1},\beta_{2},\cdots ,\beta_{L2}\right],$$

where $\alpha_{i}$ and $\beta_{i}$ represent the 121D vector.

If each amino acid sequence is encoded into a vector representation sequence, called $P_{1}$ and $P_{2}$, we created an SSA mechanism to calculate the similarity between two amino acid sequences. Based on their embedded vectors, the similarity between the two sequences was determined as follows:

(3) $$\hat{\omega }=-\frac{1}{W}\overset{L1}{\underset{i=1}{\sum }}\overset{L2}{\underset{j=1}{\sum }}\tau_{\mathrm{ij}}\|\alpha_{i}-\beta_{j}\|{}_{1}$$

$\tau_{\mathrm{ij}}$ is calculated by the following Formulas (4)–(7)

(4) $$\rho_{\mathrm{ij}}=\frac{\exp\left(-\|\alpha_{k}-\beta_{j}\|{}_{1}\right)}{\sum_{k=1}^{L1}\exp\left(-\|\alpha_{k}-\beta_{j}\|{}_{1}\right)}$$

(5) $$\sigma_{\mathrm{ij}}=\frac{\exp\left(-\|\alpha_{i}-\beta_{k}\|{}_{1}\right)}{\sum_{k=1}^{L2}\exp\left(-\|\alpha_{i}-\beta_{k}\|{}_{1}\right)}$$

(6) $$\tau_{\mathrm{ij}}=\sigma_{\mathrm{ij}}+\rho_{\mathrm{ij}}-\rho_{\mathrm{ij}}\sigma_{\mathrm{ij}}$$

(7) $$W=\overset{L1}{\underset{i=1}{\sum }}\overset{L2}{\underset{j=1}{\sum }}\tau_{\mathrm{ij}}$$

A completely differentiated SSA reversely matched these parameters to the sequence encoder parameters. Individual peptide sequence was transformed into an embedding matrix, $R^{L\times 121}$, using the trained model. A 121D SSA feature vector was produced by averaging pooling procedures.

#### 2.3.2. Pretrained BERT Embedding Model

BERT is a powerful natural language processing-inspired deep learning method [31]. The core of BERT is a transformer language model which has a variable number of encoder layers and self-attention heads, as shown in Figure 1. It provides a pretraining and fine-tuning approach, using enormous amounts of unlabeled data [32,33]. 

Here, the traditional BERT architecture was used to construct a BERT-based peptide prediction model (Figure 1) There is no need to systematically design and select feature encodings in advance. Peptide sequences were taken as input directly and passed on to the BERT method to generate feature descriptors automatically. First, the peptide sequences were converted into the token representation of k-mers as input, and the positional embedding was added to obtain the final input token. Then, the semantics of the context was captured through the multi-head self-attention model. Certain adjustments were made through linear transformation, thus ending the forward propagation of the first layer (as shown in Figure 1) There are 12 such layers in the model. The result was used for the pretraining task of BERT. The mask task is still the traditional method, covering the part and then predicting, and backpropagating through the cross-entropy loss function. A 768D BERT feature vector was produced by the BERT-trained model.

#### 2.3.3. Feature Fusion

To obtain the most superior feature combination, the 121D SSA eigenvector was combined with the 768D BERT eigenvector, which generated the 889D SSA+BERT fusion feature vector. 

#### 2.3.4. Synthetic Minority Oversampling Technique (SMOTE)

SMOTE is also called the “artificial minority oversampling method”. It is an improved scheme based on the random oversampling algorithm [39]. The random oversampling algorithm generates additional minority samples through adopting a simply copying samples strategy. As a result, it has the risk of model overfitting, where the feature information is too specific and not general enough. The SMOTE method can effectively achieve the class balance in training data [43]. The basic idea is to analyze the minority samples, synthesize new categories of samples accordingly, and add artificially simulated new samples to the dataset. Briefly, the sampling nearest neighbor algorithm calculates the KNN of each minority class sample [43]. N samples are randomly selected from K neighbors for random linear interpolation to construct new minority class samples. Combination was made subsequently between the new samples and the original data to create a new training set. The program is kept running until the data imbalance meets the relevant requirements. 

### 2.4. Machine Learning Methods 

Five commonly used high-performance ML models were used for modeling. 

The k-nearest neighbor algorithm (KNN) model [25] is to find the K sample that is most similar as the given new sample, or the K sample that is “closest to it”. If most of the K samples belong to a certain class, the sample also belongs to the same class.

Logistic regression (LR) [27] is a generalized linear model. It uses the sigmoid function to simulate the data distribution and act as the dividing line between positive and negative samples.

The support vector machine (SVM) [28,29] is to find a segmentation curve that maximizes the closest distance (also known as the interval) between data points of different classes. For binary classification, SVM is to get the furthest classification boundary and to make sure that the slight deviation of data would not have much impact.

Random forest (RF) [26] is an ensemble learning algorithm. It uses the samples with retractable samples to train multiple decision trees. Each node of the training decision tree only uses the partial features of the sampling, and it votes with the prediction results of these trees during the prediction. The voted majority class of a sample is the class to which the sample belongs.

Lighting gradient boosting machine (LGBM) [38] adopts the histogram algorithm. It converts continuous floating-point features into k discrete values, and constructs the histogram with a width of k. Then, the training data are traversed and the cumulative statistics of each discrete value in the histogram are collected. It uses a depth-limited leaf-wise strategy and supports parallel computing.

### 2.5. Performance Evaluation

Six widely used binary classification metrics were applied for performance evaluation, which are ACC, MCC, Sn, specificity (Sp), and BACC [44,45,46,47,48]. Here, TP is the given true positive sample number of umami peptides. TN is the true negative sample number of non-umami peptides. FP is the false positive sample number of non-umami peptides. FN is the false negative sample number of umami peptides.

(8) $$\mathrm{ACC}=\frac{\mathrm{TP}+\mathrm{TN}}{\mathrm{TP}+\mathrm{TN}+\mathrm{FP}+\mathrm{FN}}$$

(9) $$\mathrm{MCC}=\frac{\mathrm{TP}\times \mathrm{TN}-\mathrm{FP}\times \mathrm{FN}}{(\sqrt{\left(\mathrm{TP}+\mathrm{FP}\right)\times \left(\mathrm{TP}+\mathrm{FN}\right)\times \left(\mathrm{TN}+\mathrm{FP}\right)\times \left(\mathrm{TN}+\mathrm{FN}\right)}}$$

(10) $$\mathrm{Sn}=\frac{\mathrm{TP}}{\mathrm{TP}+\mathrm{FN}}$$

(11) $$\mathrm{Sp}=\frac{\mathrm{TN}}{\mathrm{TN}+\mathrm{FP}}$$

(12) $$\mathrm{BACC}=\frac{\mathrm{Sn}+\mathrm{Sp}}{2}$$

The receiver operating characteristic curve (ROC) is a curve drawn according to a series of different classification methods (boundary value or decision threshold), with the true positive rate (sensitivity) as the ordinate and false positive rate (specificity) as the abscissa. ROC displays the relationship between true positives and false positives at different confidence levels [12,35,49]. Nevertheless, the ROC curve cannot clearly indicate which classifier is more superior. Thus, the area under the receiver operating characteristic curve (auROC) is usually adopted as an additional metric for model evaluation. The classifier with a larger auROC value performs better. The value of auROC for proposed models was computed and used to compare with the models reported previously.

For the model evaluation method, the widely used K-fold cross-validation method and independent testing method were adopted. Firstly, the K-fold cross-validation were applied for model training and validation evaluation based on the training set. In this study, the K value was 10. That is, the training set was randomly divided into ten parts, of which nine were used for training and one for validation. The performance of the trained model was evaluated by the average of 10 validation scores. Independent testing was to use additional new data, not in the training set, to test and evaluate the trained model. A good model requires good metrics value for both K-fold cross-validation and independent testing.

## 3. Results and Discussion

### 3.1. Preliminary Performance of Models Trained with or without SMOTE

**Figure 2:**
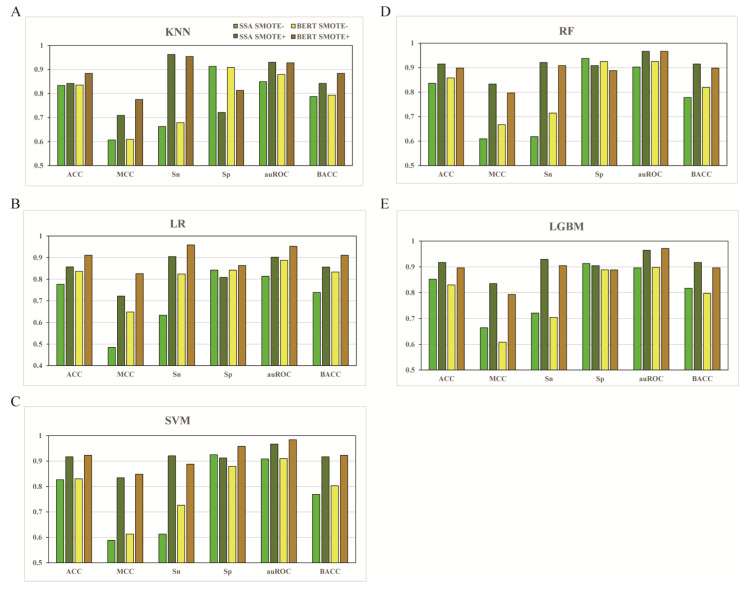
The performance of 10-fold cross-validation metrics of SSA and BERT features using different algorithms pretrained with or without SMOTE. (**A**) KNN; (**B**) LR; (**C**) SVM; (**D**) RF; (**E**) LGBM.

To overcome the data imbalance in modeling, the SMOTE method was first applied to the modeling. Meanwhile, to explore the embedding feature types in umami peptides, different models were built based on two deep representation learning feature extraction methods, the pretrained SSA embedding model and the pretrained BERT embedding model, in combination with five distinct widely-used ML algorithms (KNN, LR, SVM, RF, and LGBM) The performance of the different combination models pretrained with or without SMOTE was compared by performing the repeated stratified 10-fold cross validation tests 10 times (Figure 2)

For 10-fold cross-validation results (Figure 2), all five algorithm models using the SMOTE method based on either the SSA or BERT feature performed better across five metrics (ACC, MCC, Sn, auROC, and BACC) than the models not using SMOTE, with Sp as the exception. The scores after model parameter optimization are listed in Table 1. For example, the average ACCs of KNN, LR, SVM, RF, and LGBM based on SSA with SMOTE are 0.842, 0.857, 0.917, 0.915, and 0.917, respectively, which exceeded that of the models without SMOTE by 1.08%, 10.44%, 10.88%, 9.45%, and 7.63%, respectively. A similar improvement was also observed in the 10-fold cross-validation results based on the BERT feature (Figure 2 and Table 1) Although the best Sp values based on the SSA feature with SMOTE (0.913) were lower than those of the model without SMOTE (0.938), the overall best Sp score (0.959) was still obtained from the BERT feature optimized using the SMOTE method. For SMOTE performance in the independent test of the SSA or BERT feature vector (Table 1), still, the best scores were achieved using the SMOTE method across the five metrics. Take values based on SSA for example, the ACC is 0.866, with MCC to be 0.683, Sn to be 0.814, auROC to be 0.916, and BACC to be 0.825. These results indicate that increasing the sampling with SMOTE could effectively overcome the data imbalance and improve model performance in predicting umami peptides. Particularly, we noted that the BACC scores based on the five algorithms in the cross-validation results were the same as ACC with SMOTE being used (Figure 2 and Table 1) As the metric BACC reflected the level of data balance, the data became balanced after SMOTE application, and BACC became redundant. Similar results were observed in the subsequent cross-validation analysis with SMOTE.

### 3.2. The Effect of Different Feature Types

Meanwhile, from the cross-validation results (Figure 2 and Table 1), the BERT feature vector developed using the SVM algorithm with SMOTE method performed best out of all the combinations tested across the five metrics (ACC, MCC, Sp, auROC, and BACC) Among them, ACC was 0.923 (0.65–18.9%) higher than the other options, with MCC being 0.849 higher by 1.67–75.0%, Sp being 0.959 higher by 2.24–33.0%, auROC being 0.884 higher by 1.76–20.9%, and BACC being 0.923 higher by 0.65–20.0%. Nevertheless, the SSA feature vector conjugated with KNN and SMOTE algorithms outperformed all the BERT combinations across the Sn metric (0.962) Regarding the performance of the BERT feature vector based on SVM with SMOTE in the independent test (Table 1), ACC was 0.876 lower by 2.03% compared with that of the BERT feature based on RF using SMOTE, with MCC being 0.706 lower by 11.0%, Sn being 0.714 lower by 21.1%, Sp being 0.951 higher by 7.09%, auROC being 0.926 lower by 4.63%, and BACC being 0.832 lower by 7.24%. Yet, the BERT-SVM-SMOTE combination was still supposed to be the best model out of all the combinations.

### 3.3. The Effect of Feature Fusion

**Figure 3:**
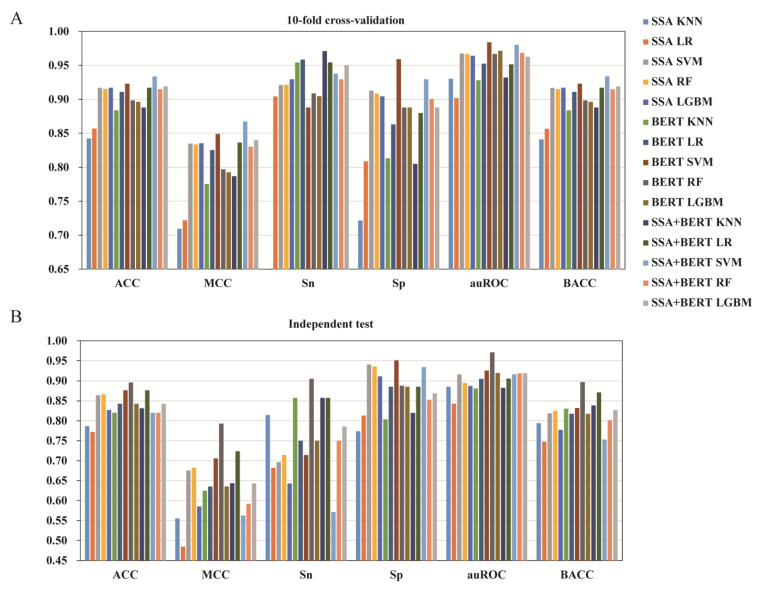
The performance metrics of individual and fused features with SMOTE, according to the machine learning methods used. (**A**) Ten-fold cross-validation results. (**B**) Independent test results.

To further improve the model performance and obtain more information, the SSA and BERT features were combined to make fusion features. The fusion feature was combined with the five algorithms (KNN, LR, SVM, RF, and LGBM) to train baseline models and improve model performance. Table 2 displayed the 10-fold cross-validation and independent testing results of the SSA-BERT fusion features with or without SMOTE. The performance metrics of the individual and fused features with SMOTE according to the ML methods are summarized in Figure 3. Consistent with the results in Section 3.1, for the 10-fold cross-validation (Table 2), the SSA-BERT fusion feature with five models using SMOTE displayed a remarkably higher value than the models without SMOTE except for the Sp value, and the BACC score was the same as ACC with SMOTE being used. Particularly, the best performance of the fusion feature was slightly superior to the BERT feature alone across four metrics, with ACC being 0.934 higher by 1.19%, MCC being 0.867 higher by 1.90%, Sn being 0.971 higher by 1.25%, and BACC being 0.934 higher by 1.19%. However, the best performance of the fusion feature in the independent test results across all the six metrics (ACC = 0.876, MCC = 0.724, Sn = 0.857, Sp = 0.934, auROC = 0.919, BACC = 0.871) was in any aspect lower than the corresponding scores in the BERT feature alone (ACC = 0.896, MCC = 0.793, Sn = 0.905, Sp = 0.951, auROC = 0.971, BACC = 0.897) with SMOTE (Figure 3 and Table 2) Thus, the feature fusion of SSA and BERT is not a beneficial choice for model optimization in umami peptide automatic prediction.

### 3.4. The Effect of Feature Selection

**Figure 4:**
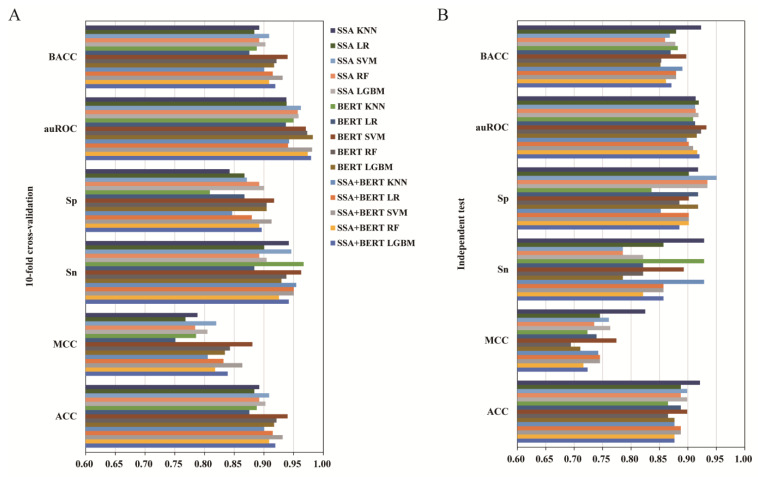
The performance metrics of individual and fusion features using selected features and different algorithms. (**A**) Ten-fold cross-validation results. (**B**) Independent test results.

As described in Section 3.3, feature fusion was not superior to BERT feature alone. In the training set, the sequence vector had 121 dimensions based on SSA feature, and 768 dimensions based on BERT, respectively. The feature vectors had 889 dimensions based on the combined fusion feature. Higher dimensions indicated a higher risk of information redundancy, that would result in model overfitting. Feature selection is a good way to solve this problem, which removes redundant and indistinguishable features [38]. The LGBM feature selection method has been proved to an effective approach for feature selection and was successfully applied for ML-based bio-sequence classification [38,50]. Here, we also used it to find the optimized feature space for umami peptide prediction task. Table 3 presented the performance metrics of the individual and fused features created based on five ML models (KNN, LR, SVM, RF, and LGBM) in conjugation with SMOTE. A visual illustration of the outcomes was shown in Figure 4. 

From the 10-fold cross-validation results (Figure 4 and Table 3), using feature selection, all the individual or fusion features based on the SVM algorithm outperformed the other four algorithms (KNN, LR, RF, and LGBM) across four metrics, namely ACC, MCC, Sp, and BACC. The best performance was observed in the BERT feature encoding alone based on the SVM algorithm with 139 dimensions over all the other options (Table 3), with ACC (0.940) better by 0.86–7.31%, MCC (0.881) better by 1.97–17.31%, Sp (0.917) better by 0.44–13.35%, and BACC (0.940) better by 0.86–7.31%. These results indicate that selecting a feature descriptor is an effective method for optimizing the performance of the umami peptide prediction model. For the independent test (Table 3), although the highest scores of ACC (0.921), MCC (0.825), Sn (0.929), Sp (0.951), and BACC (0.923) were obtained based on the SSA feature either in conjugation with KNN (43 dimensions) or SVM (29 dimensions), yet the BERT feature still performed better than the other options across the auROC (0.933) metric based on SVM with 139 dimensions. Additionally, the scores of the other four metrics based on BERT, namely ACC (0.899), MCC (0.774), Sn (0.893), and BACC (0.897), were the second best among all the models. The results of both cross-validation and independent testing suggest that the BERT feature based on the SVM algorithm (139D) was the best option for umami peptide prediction.

### 3.5. Comparison of iUP-BERT with Existing Models

The efficacy and robustness of the iUP-BERT model in umami peptide identification was evaluated subsequently. Its predictive performance was compared with that of the existing methods, namely iUmami-SCM and UMPred-FRL. As shown in Table 4, from the cross-validation results, iUP-BERT apparently outperformed iUmami-SCM and UMPred-FRL across ACC, MCC, Sn, auROC, and BACC. Regarding the independent test results, iUP-BERT produced remarkably better results in the five metrics than iUmami-SCM and UMPred-FRL; for ACC by 1.23–3.93%, for MCC by 5.31–13.99%, for Sn by 13.6–25.07%, for auROC by 1.52–3.90%, and for BACC by 4.30–8.86%. Taken together, the comparisons show that iUP-BERT based on the BERT-SVM-SMOTE combination is more effective, reliable, and stable than the existing methods for umami peptide prediction.

### 3.6. Feature Analysis Using Feature Projection and Decision Function 

**Figure 5:**
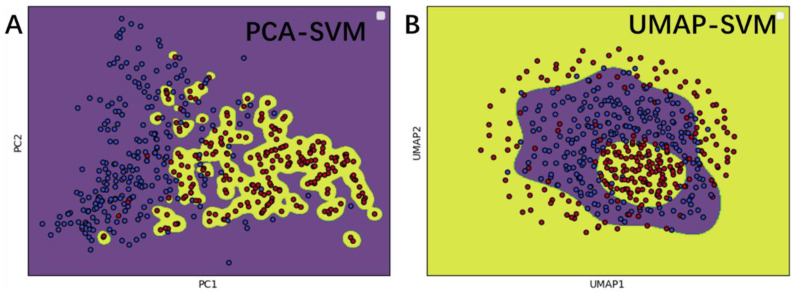
Dimension reduction visualization of umami peptide BERT features and decision function boundary analysis of the SVM model. The red dots are umami peptides and the blue dots are non-umami peptides. The sub-figure (**A**,**B**) show the use of principal components analysis (PCA) and uniform manifold approximation and projection (UMAP) respectively for reducing 139 dimensional selected BERT features to 2 dimensions for visual analysis. Additionally, the decision function boundary lines of support vector machine (SVM) are drawn in both. The yellow section represents the positive sample area and the purple section represents the negative sample area.

To visually explain the excellent performance of iUP-BERT, principal components analysis (PCA) and uniform manifold approximation and projection (UMAP) dimension reduction were used. First, the feature space vector optimized by feature selection, namely BERT features of 139D, was reduced to a 2-dimensional plane using PCA and UAMP algorithms, respectively. As displayed in Figure 5, red dots represented umami peptides and blue dots represented non-umami peptides. Then, a decision function boundary was drawn, which could distinguish between positive and negative samples. As shown in Figure 5, the distribution of positive and negative samples is relatively concentrated in two areas; the positive samples are most in yellow areas, while the negative samples in the purple area. Additionally, we can see from Figure 5, that SVM can distinguish most positive and negative samples, yet there are still some misclassified samples. Therefore, better feature extraction methods or more suitable machine learning methods were needed for modeling, to better identify umami peptide sequences from non-umami peptide sequences in the future.

### 3.7. Construction of the Web Server of iUP-BERT

To facilitate rapid and high-throughput screening of umami peptides and maximize the use of the iUP-BERT predictor, an open-access web server was established at https://www.aibiochem.net/servers/iUP-BERT/ (accessed on 23 September 2022) We hope the iUP-BERT would be a powerful tool that can be used to explore new umami peptides and to promote the food seasoning industry.

## 4. Conclusions

In this study, a novel machine learning prediction model, namely iUP-BERT, was developed for the accurate prediction of umami peptides based on the peptide sequence alone. A single deep representation learning feature encoding method (BERT) was adopted to generate predicted probabilistic scores of potential umami peptides. First, SMOTE was applied to balance the data. Then, feature extraction approaches (SSA, BERT, or fused feature) were combined with five different algorithms (KNN, LR, SVM, RF, and LGBM) to build different models. After extensive testing and optimization, the BERT-SVM-SMOTE model with 139 dimensions was the best feature set. Further feature selection produced a robust model. To our knowledge, this is the first report on the utilization of the deep representing learning feature BERT in the computational identification of umami peptides. Subsequent 10-fold cross-validation and independent test results indicated the efficacy and robustness of iUP-BERT in predicting umami peptides. By comparison with the existing methods (iUmami-SCM and UMPred-FRL) based on the independent test, the iUP-BERT with BERT feature extraction method alone significantly outperformed the existing predictors with several manual feature extraction combinations; for ACC by 1.23–3.93%, for MCC by 5.31–13.99%, for Sn by 13.6–25.07%, for auROC by 1.52–3.90%, and for BACC higher by 4.30–8.86%. Finally, to maximize the use of the predictor, an open-access iUP-BERT web server was built at https://www.aibiochem.net/servers/iUP-BERT/ (accessed on 23 September 2022) For deep learning-based models, larger training sample size improves the prediction performance. As the number of the training datasheet used here were relatively low (112 positive and 241 negative samples), future efforts could be exerted on constructing an optimized larger size datasheet with higher amounts of identified umami and non-umami peptides for better model performance. Additionally, it would be to achieve a more accuracy model by fine-tuning the BERT for feature extraction. Finally, we hope the iUP-BERT would be a powerful tool for exploring new umami peptides to promote the umami seasoning industry.

## Figures and Tables

**Table 1:** Performance metrics of two different deep representation learning features using five machine learning models with or without SMOTE.

Feature	Model	SMOTE	Dim	10-Fold Cross-Validation						Independent Test					
				ACC	MCC	Sn	Sp	auROC	BACC	ACC	MCC	Sn	Sp	auROC	BACC
SSA ^b^	KNN ^c^	−	121	0.833	0.607	0.663	0.913	0.849	0.788	0.825	0.575	0.596	0.930	0.876	0.763
	LR ^c^	−	121	0.776	0.485	0.634	0.842	0.814	0.738	0.780	0.498	0.679	0.826	0.839	0.752
	SVM ^c^	−	121	0.827	0.588	0.613	0.925	0.909	0.769	0.857	0.658	0.668	0.944	0.907	0.806
	RF ^c^	−	121	0.836	0.609	0.618	0.938	0.902	0.778	0.826	0.578	0.557	0.949	0.879	0.753
	LGBM ^c^	−	121	0.852	0.664	0.721	0.913	0.896	0.817	0.827	0.583	0.621	0.921	0.880	0.771
	KNN ^c^	+	121	0.842	0.709	**0.962** ^a^	0.721	0.930	0.841	0.787	0.555	0.814	0.774	0.885	0.794
	LR ^c^	+	121	0.857	0.722	0.904	0.809	0.902	0.856	0.772	0.485	0.682	0.813	0.843	0.748
	SVM ^c^	+	121	0.917	0.835	0.921	0.913	0.967	0.917	0.864	0.675	0.696	0.941	0.916	0.819
	RF ^c^	+	121	0.915	0.833	0.921	0.908	0.967	0.915	0.866	0.683	0.714	0.936	0.895	0.825
	LGBM ^c^	+	121	0.917	0.835	0.929	0.904	0.964	0.917	0.827	0.585	0.643	0.911	0.887	0.777
BERT ^b^	KNN ^c^	−	768	0.836	0.610	0.679	0.908	0.879	0.794	0.807	0.537	0.618	0.893	0.872	0.756
	LR ^c^	−	768	0.836	0.649	0.824	0.842	0.888	0.833	0.855	0.660	0.743	0.907	0.912	0.825
	SVM ^c^	−	768	0.830	0.613	0.727	0.880	0.910	0.803	0.820	0.599	0.775	0.841	0.875	0.808
	RF ^c^	−	768	0.859	0.667	0.714	0.925	0.925	0.820	0.819	0.567	0.643	0.900	0.900	0.771
	LGBM ^c^	−	768	0.830	0.609	0.705	0.889	0.898	0.797	0.830	0.596	0.668	0.905	0.915	0.786
	KNN ^c^	+	768	0.884	0.775	0.954	0.813	0.928	0.884	0.820	0.625	0.857	0.803	0.881	0.830
	LR ^c^	+	768	0.911	0.825	0.959	0.863	0.952	0.911	0.843	0.635	0.750	0.885	0.905	0.818
	SVM ^c^	+	768	**0.923**	**0.849**	0.888	**0.959**	**0.984**	**0.923**	0.876	0.706	0.714	**0.951**	0.926	0.832
	RF ^c^	+	768	0.898	0.797	0.909	0.888	0.967	0.898	**0.896**	**0.793**	**0.905**	0.888	**0.971**	**0.897**
	LGBM ^c^	+	768	0.896	0.793	0.905	0.888	0.971	0.896	0.843	0.635	0.750	0.885	0.920	0.818

^a^ Best performance values are in bold and are underlined. ^b^ SSA: soft symmetric alignment; BERT: bidirectional encoder representations from transformer. ^c^ KNN: k-nearest neighbor; LR: logistic regression; SVM: support vector machine; RF: random forest. LGBM: light gradient boosting machine. “-” indicates without the SMOTE method; “+” indicates with the SMOTE method.

**Table 2:** Performance metrics of fusion features using five machine learning models with or without SMOTE.

Feature	Model	SMOTE	Dim	10-Fold Cross-Validation						Independent Test					
				ACC	MCC	Sn	Sp	auROC	BACC	ACC	MCC	Sn	Sp	auROC	BACC
SSA ^b^ + BERT ^b^	KNN ^c^	−	889	0.836	0.610	0.679	0.909	0.908	0.794	0.820	0.576	0.679	0.885	0.900	0.782
	LR ^c^	−	889	0.844	0.640	0.750	0.888	0.900	0.819	**0.876** ^a^	0.716	0.821	0.902	0.910	0.862
	SVM ^c^	−	889	0.858	0.667	0.732	0.917	0.921	0.825	0.854	0.658	0.750	0.902	0.906	0.826
	RF ^c^	−	889	0.841	0.620	0.643	**0.934**	0.906	0.788	0.831	0.599	0.679	0.902	0.906	0.790
	LGBM ^c^	−	889	0.813	0.553	0.625	0.900	0.892	0.763	0.831	0.606	0.714	0.885	0.921	0.800
	KNN ^c^	+	889	0.888	0.787	**0.971**	0.805	0.932	0.888	0.831	0.643	**0.857**	0.820	0.883	0.838
	LR ^c^	+	889	0.917	0.836	0.954	0.880	0.951	0.917	0.876	**0.724**	**0.857**	0.885	0.906	**0.871**
	SVM ^c^	+	889	**0.934**	**0.867**	0.938	0.929	**0.980**	**0.934**	0.820	0.563	0.571	**0.934**	0.916	0.753
	RF ^c^	+	889	0.915	0.830	0.929	0.900	0.968	0.915	0.820	0.592	0.750	0.852	**0.919**	0.801
	LGBM ^c^	+	889	0.919	0.840	0.950	0.888	0.963	0.919	0.843	0.643	0.786	0.869	**0.919**	0.827

^a^ Best performance values are in bold and are underlined. ^b^ SSA: soft symmetric alignment; BERT: bidirectional encoder representations from transformer. ^c^ KNN: k-nearest neighbor; LR: logistic regression; SVM: support vector machine; RF: random forest. LGBM: light gradient boosting machine. “-” indicates without the SMOTE method; “+” indicates with the SMOTE method.

**Table 3:** Performance metrics of individual and fused features according to the machine learning methods.

Feature	Model	SMOTE	Dim	10-Fold Cross-Validation						Independent Test					
				ACC	MCC	Sn	Sp	auROC	BACC	ACC	MCC	Sn	Sp	auROC	BACC
SSA ^b^	KNN ^c^	+	43	0.892	0.788	0.942	0.842	0.938	0.892	**0.921** ^a^	**0.825**	**0.929**	0.918	0.914	**0.923**
	LR ^c^	+	41	0.884	0.768	0.900	0.867	0.938	0.884	0.888	0.745	0.857	0.902	0.919	0.879
	SVM ^c^	+	29	0.909	0.820	0.946	0.871	0.962	0.909	0.899	0.761	0.786	**0.951**	0.913	0.868
	RF ^c^	+	30	0.892	0.784	0.892	0.892	0.957	0.892	0.888	0.735	0.786	0.934	0.914	0.860
	LGBM ^c^	+	39	0.902	0.805	0.905	0.900	0.958	0.902	0.899	0.763	0.821	0.934	0.919	0.878
BERT ^b^	KNN ^c^	+	163	0.888	0.786	**0.967**	0.809	0.950	0.888	0.865	0.723	**0.929**	0.836	0.909	0.882
	LR ^c^	+	29	0.876	0.751	0.884	0.867	0.937	0.876	0.888	0.739	0.821	0.918	0.913	0.870
	SVM ^c^	+	139	**0.940**	**0.881**	0.963	**0.917**	0.971	**0.940**	0.899	0.774	0.893	0.902	**0.933**	0.897
	RF ^c^	+	79	0.921	0.843	0.938	0.905	0.973	0.921	0.865	0.694	0.821	0.885	0.923	0.853
	LGBM ^c^	+	174	0.917	0.834	0.929	0.905	**0.982**	0.917	0.876	0.711	0.786	0.918	0.916	0.852
SSA ^b^ + BERT ^b^	KNN ^c^	+	65	0.900	0.806	0.954	0.846	0.942	0.900	0.876	0.742	**0.929**	0.852	0.898	0.891
	LR ^c^	+	74	0.915	0.832	0.950	0.880	0.941	0.915	0.888	0.745	0.857	0.902	0.902	0.879
	SVM ^c^	+	39	0.932	0.864	0.950	0.913	0.981	0.932	0.888	0.745	0.857	0.902	0.909	0.879
	RF ^c^	+	168	0.909	0.818	0.925	0.892	0.974	0.909	0.876	0.716	0.821	0.902	0.917	0.862
	LGBM ^c^	+	114	0.919	0.839	0.942	0.896	0.979	0.919	0.876	0.724	0.857	0.885	0.920	0.871

^a^ Best performance values are in bold and are underlined. ^b^ SSA: soft symmetric alignment; BERT: bidirectional encoder representations from transformer. ^c^ KNN: k-nearest neighbor; LR: logistic regression; SVM: support vector machine; RF: random forest. LGBM: light gradient boosting machine. “+” indicates with the SMOTE method.

**Table 4:** Cross-validation and independent test results of iUP-BERT and the existing methods.

Classifier	10-Fold Cross-Validation						Independent Test					
	ACC	MCC	Sn	Sp	auROC	BACC	ACC	MCC	Sn	Sp	auROC	BACC
iUP-BERT	**0.940** ^a^	**0.881**	**0.963**	0.917	**0.971**	**0.940**	**0.899**	**0.774**	**0.893**	0.902	**0.933**	**0.897**
iUmami-SCM	0.935	0.864	0.947	0.930	0.945	0.939	0.865	0.679	0.714	**0.934**	0.898	0.824
UMPred-FRL	0.921	0.814	0.847	**0.955**	0.938	0.901	0.888	0.735	0.786	**0.934**	0.919	0.860

^a^ Best performance values are in bold and are underlined.

## Data Availability

The data used to support the findings of this study can be made available by the corresponding author upon request.

## Supplementary Materials

The following supporting information can be downloaded at: https://www.mdpi.com/article/10.3390/foods11223742/s1, File S1: Datasets for training and independent test.

## Abbreviation

The following abbreviations are used in this manuscript:

ML	machine learning
BERT	bidirectional encoder representations from transformer
SSA	soft symmetric alignment
SMOTE	synthetic minority over-sampling technique
KNN	k-nearest neighbor
RF	random forest
SVM	support vector machine
LGBM	light gradient boosting machine
LR	logistic regression
SCM	scoring card method
ACC	accuracy
BACC	deduced balanced accuracy
Sn	sensitivity
Sp	specificity
MCC	Matthew’s coefficient correlation
ROC	receiver operating characteristic curve
auROC	area under the receiver operating characteristic curve
PCA	principal components analysis
UMAP	uniform manifold approximation and projection
