# Supplementary material for: IUP-BERT: Identification of Umami Peptides Based on BERT Features
Source: Foods. 2022 Nov 21;11(22):3742. doi: 10.3390/foods11223742 (PMC9689418; doi:10.3390/foods11223742)
Supplement: Supplementary file 1 [file foods-11-03742-s001.zip › foods-1959144-supplementary.pdf]

File S1: Datasets for training and independent test

Training dataset for positive sample

>Positive_0
RKE
>Positive_1
VAV
>Positive_2
KGDEESLA
>Positive_3
SAEQK
>Positive_4
CM
>Positive_5
DG
>Positive_6
DE
>Positive_7
EEL
>Positive_8
LVG
>Positive_9
SLAKGDEE
>Positive_10
DD
>Positive_11
VGG
>Positive_12
EEE
>Positive_13
LSERYP
>Positive_14
GCG
>Positive_15
AHSVRFY
>Positive_16
EEEQ
>Positive_17
YGGTPPFV
>Positive_18
EN
>Positive_19

LPEEV
>Positive_20
ASNMSDL
>Positive_21
EINEL
>Positive_22
EE
>Positive_23
SE
>Positive_24
GDG
>Positive_25
VDV
>Positive_26
PVARMCR
>Positive_27
VTADESQQDVLK
>Positive_28
VV
>Positive_29
DPQ
>Positive_30
EQEEL
>Positive_31
HCHTN
>Positive_32
VVGET
>Positive_33
NVVGET
>Positive_34
ENINEL
>Positive_35
SF
>Positive_36
LV
>Positive_37
DEE
>Positive_38
EA
>Positive_39
INEL
>Positive_40
SEE
>Positive_41

AQALQAQA
>Positive_42
LQPLNAH
>Positive_43
KGRYER
>Positive_44
GLLPDGTPR
>Positive_45
DES
>Positive_46
PDKPNT
>Positive_47
EGS
>Positive_48
EP
>Positive_49
CCNKSV
>Positive_50
LGAGGSLA
>Positive_51
QVAIAHRDAK
>Positive_52
DQR
>Positive_53
ALPEEV
>Positive_54
GGY
>Positive_55
EQQQQ
>Positive_56
VE
>Positive_57
SPE
>Positive_58
ET
>Positive_59
ENE
>Positive_60
VD
>Positive_61
PET
>Positive_62
KGNEESLA
>Positive_63

EDV
>Positive_64
DA
>Positive_65
ANPGPVRDLR
>Positive_66
QL
>Positive_67
RGENESDEQGAIVT
>Positive_68
EG
>Positive_69
AE
>Positive_70
NNP
>Positive_71
VRSY
>Positive_72
LDL
>Positive_73
RPNPFENR
>Positive_74
NGKET
>Positive_75
EK
>Positive_76
SSRNEQSR
>Positive_77
EQE
>Positive_78
RGENESEEEGAIVT
>Positive_79
DDD
>Positive_80
EGSEAPDGSSR
>Positive_81
NRTF
>Positive_82
PE
>Positive_83
NY
>Positive_84
KGSLADEE
>Positive_85

VPY
>Positive_86
EL
>Positive_87
EY
>Positive_88
RPLGNC
>Positive_89
VLPTDQNFILR
>Positive_90
DDE
>Positive_91
VEV
>Positive_92
RL
>Positive_93
SLADEEKG
>Positive_94
ADE
>Positive_95
KG
>Positive_96
EDD
>Positive_97
ADA
>Positive_98
EDF
>Positive_99
TE
>Positive_100
QEEL
>Positive_101
ES
>Positive_102
GEG
>Positive_103
PAA
>Positive_104
EDE
>Positive_105
EEDGK
>Positive_106
AD
>Positive_107

VDR
>Positive_108
DDDD
>Positive_109
GE
>Positive_110
ED
>Positive_111
TLRRCM

Training dataset for negative sample

>Negative_0
EQPQQNE
>Negative_1
QDKIHPFAQTQSLVYPFPGP
>Negative_2
GRP
>Negative_3
FGG
>Negative_4
LAGNQEEE
>Negative_5
APK
>Negative_6
KPF
>Negative_7
GGLGG
>Negative_8
PGI
>Negative_9
AF
>Negative_10
YY
>Negative_11
IG
>Negative_12
FGGF
>Negative_13
RPGFF
>Negative_14
FGF
>Negative_15

QSKVLPVPQ
>Negative_16
GNPDIEHP
>Negative_17
VYPFPPGIGG
>Negative_18
GF
>Negative_19
IV
>Negative_20
RG
>Negative_21
FLL
>Negative_22
GPPF
>Negative_23
FYPELF
>Negative_24
KLHENIAR
>Negative_25
GPG
>Negative_26
LA
>Negative_27
YYY
>Negative_28
LGGGG
>Negative_29
GGP
>Negative_30
YL
>Negative_31
QLFGPNVNPWHNP
>Negative_32
RGPPFFF
>Negative_33
RGPPF
>Negative_34
VIFPPGR
>Negative_35
PK
>Negative_36
YGG
>Negative_37

VVVPPFLQP
>Negative_38
VL
>Negative_39
YPFPGPIHNS
>Negative_40
GGRPFF
>Negative_41
YQEPVLGPVRGPFPI
>Negative_42
PFPGPIP
>Negative_43
PPF
>Negative_44
AQTQSLVYPFPGPIPNSLPQNIPPLTQ
>Negative_45
RRPPPFFF
>Negative_46
RGPGPIIV
>Negative_47
RGPPFI
>Negative_48
RPF
>Negative_49
YG
>Negative_50
ALEPDHR
>Negative_51
VPPFLE
>Negative_52
RPGGFF
>Negative_53
PGPGPG
>Negative_54
GGF
>Negative_55
VIPFPGR
>Negative_56
LGL
>Negative_57
RPFG
>Negative_58
LL
>Negative_59

FF
>Negative_60
RP
>Negative_61
QLFGPNVNPWHNP
>Negative_62
KPK
>Negative_63
RGPPGFF
>Negative_64
AV
>Negative_65
PVLGPVRGPFPIIV
>Negative_66
VYPFPPGI
>Negative_67
YQEPVLGPVRGPFPIIV
>Negative_68
RGPPGGFF
>Negative_69
RGPEPIIV
>Negative_70
RGPPFIIV
>Negative_71
QPVLGPVRGPFPIIV
>Negative_72
GY
>Negative_73
VIF
>Negative_74
DY
>Negative_75
VYPFPPGINH
>Negative_76
RPKHPIKHQ
>Negative_77
EPAD
>Negative_78
FG
>Negative_79
SL
>Negative_80
MPFPKYPVEPF
>Negative_81

QLFNPSTNPWHSP
>Negative_82
GPVRGPFPIIV
>Negative_83
YQQPVLGPVRGPFPI
>Negative_84
RRPPGF
>Negative_85
YQQPVLGPVRGPFPII
>Negative_86
RGFF
>Negative_87
VVVPPFL
>Negative_88
YPFPGPI
>Negative_89
KAVPYPPQ
>Negative_90
VPLGTQYTDAPSF
>Negative_91
QLFNPSTNP
>Negative_92
APFPEVFG
>Negative_93
PL
>Negative_94
LF
>Negative_95
QNDKIHPFAQTQSLVYPFGPIP
>Negative_96
PG
>Negative_97
FPF
>Negative_98
LRL
>Negative_99
FFG
>Negative_100
PI
>Negative_101
QLFNPS
>Negative_102
GP
>Negative_103

PGPIP
>Negative_104
LI
>Negative_105
FIV
>Negative_106
GPFPII
>Negative_107
LG
>Negative_108
PF
>Negative_109
GGGLG
>Negative_110
GFF
>Negative_111
GGV
>Negative_112
GV
>Negative_113
YGY
>Negative_114
RGPPGIG
>Negative_115
VYPF
>Negative_116
SAEFG
>Negative_117
YLGYLEQLLR
>Negative_118
RGPPGGGFF
>Negative_119
YF
>Negative_120
YEGNS
>Negative_121
GGRGPPFIV
>Negative_122
IT
>Negative_123
FY
>Negative_124
QQPVLGPVRGPFPI
>Negative_125

PFPGPI
>Negative_126
GL
>Negative_127
VYP
>Negative_128
AYFYPEL
>Negative_129
FL
>Negative_130
GPFPVI
>Negative_131
NFNNQLDQQTPR
>Negative_132
RRR
>Negative_133
PFP
>Negative_134
FFPP
>Negative_135
ELL
>Negative_136
LVYPFPGPIHN
>Negative_137
GLGG
>Negative_138
LGG
>Negative_139
RGPFPIIV
>Negative_140
TDVENLHLPPLL
>Negative_141
FPP
>Negative_142
WE
>Negative_143
SLA
>Negative_144
PFIV
>Negative_145
GLY
>Negative_146
LGGG
>Negative_147

VI
>Negative_148
PPFIV
>Negative_149
VYPFPPIGNH
>Negative_150
RGP
>Negative_151
GGFFGG
>Negative_152
GR
>Negative_153
GGL
>Negative_154
GPFF
>Negative_155
RGPPGF
>Negative_156
EQGGEQG
>Negative_157
RPFFGG
>Negative_158
IK
>Negative_159
KPPFIV
>Negative_160
IP
>Negative_161
LY
>Negative_162
VY
>Negative_163
RGPPFIV
>Negative_164
IQ
>Negative_165
IW
>Negative_166
RPG
>Negative_167
WF
>Negative_168
RGPFIV
>Negative_169

LRP
>Negative_170
PPP
>Negative_171
RRPFF
>Negative_172
EEN
>Negative_173
YPF
>Negative_174
WW
>Negative_175
FV
>Negative_176
PA
>Negative_177
PPG
>Negative_178
GGGGL
>Negative_179
FALPQYLK
>Negative_180
GKHQQEEENEGG
>Negative_181
GVV
>Negative_182
PGR
>Negative_183
FW
>Negative_184
GGGL
>Negative_185
LD
>Negative_186
GGRPFFGG
>Negative_187
GLG
>Negative_188
VIFPPG
>Negative_189
IF
>Negative_190
QLFNPSTNPWH
>Negative_191

FFPGG
>Negative_192
GPFPIIV
>Negative_193
VYPFPPG
>Negative_194
RGPPGGV
>Negative_195
GLL
>Negative_196
RGPPFF
>Negative_197
RPPPFFF
>Negative_198
NALPE
>Negative_199
FPPFIV
>Negative_200
RPFFRPFFRPFF
>Negative_201
GGRGPPFIVGG
>Negative_202
YLEQLLR
>Negative_203
EVLN
>Negative_204
EGG
>Negative_205
LW
>Negative_206
LLL
>Negative_207
GGVVV
>Negative_208
GGFF
>Negative_209
NNEDT
>Negative_210
QLFNPSTNPWHSP
>Negative_211
MAPKHKEMPFPKYPVEPF
>Negative_212
KF
>Negative_213

NLQG
>Negative_214
FPK
>Negative_215
RGPFPIV
>Negative_216
RPFF
>Negative_217
IGTLAGA
>Negative_218
EI
>Negative_219
APFPEVF
>Negative_220
PP
>Negative_221
PVRGPFPIIV
>Negative_222
FFVAPFPEVFGK
>Negative_223
FP
>Negative_224
IL
>Negative_225
PVLGPV
>Negative_226
FFPR
>Negative_227
RRPPFF
>Negative_228
VVYPWTQRF
>Negative_229
GYG
>Negative_230
PY
>Negative_231
RGPPFGG
>Negative_232
YYG
>Negative_233
GFG
>Negative_234
YP
>Negative_235

LLLL
>Negative_236
GYY
>Negative_237
RPFFRPFF
>Negative_238
IE
>Negative_239
IS
>Negative_240
VA

Independent dataset for positive sample

>Positive 0
CALTP
>Positive 1
AEA
>Positive 2
VG
>Positive 3
VF
>Positive 4
AAPY
>Positive 5
VEL
>Positive 6
TESSSE
>Positive 7
TAY
>Positive 8
STMLLESER
>Positive 9
SFE
>Positive 10
PSE
>Positive 11
PAQ
>Positive 12
LYER
>Positive 13
LEQL
>Positive 14
LE

>Positive 15
HV
>Positive 16
GGNP
>Positive 17
GD
>Positive 18
EV
>Positive 19
EPE
>Positive 20
EGF
>Positive 21
EED
>Positive 22
EDG
>Positive 23
EAGIQ
>Positive 24
DL
>Positive 25
DGG
>Positive 26
DEL
>Positive 27
DED

Independent dataset for negative sample

>Negative 0
YQQPVLGPVRGPFPIIV
>Negative 1
PIP
>Negative 2
FI
>Negative 3
PGP
>Negative 4
FGFG
>Negative 5
PGG
>Negative 6
PFPP

>Negative 7
PFPIIV
>Negative 8
PFPGPIPNS
>Negative 9
FFRPFFRPFF
>Negative 10
FFPG
>Negative 11
FFPE
>Negative 12
FFGG
>Negative 13
WWW
>Negative 14
FFF
>Negative 15
WP
>Negative 16
WL
>Negative 17
NENLL
>Negative 18
NALEPDHRVE
>Negative 19
EF
>Negative 20
VYPFGGGINH
>Negative 21
DV
>Negative 22
DLL
>Negative 23
LLG
>Negative 24
VVV
>Negative 25
VPPFLQ
>Negative 26
LGYLEQLL
>Negative 27
APKHKEMPFPKYPVEPF
>Negative 28
AL

>Negative 29
VIIPFPGR
>Negative 30
VIIPFPG
>Negative 31
LEQLL
>Negative 32
AGNPDIEHPE
>Negative 33
KP
>Negative 34
RRPP
>Negative 35
RR
>Negative 36
RPPFIV
>Negative 37
IN
>Negative 38
II
>Negative 39
RPGF
>Negative 40
ID
>Negative 41
IA
>Negative 42
GW
>Negative 43
RGPPGGF
>Negative 44
RGPPGFG
>Negative 45
GPPFIV
>Negative 46
RGPPFIVRGPPFIV
>Negative 47
RGPPFIVGG
>Negative 48
GMIYPG
>Negative 49
RGPKPIIV
>Negative 50
GLGGG

>Negative 51
GI
>Negative 52
RGGFIV
>Negative 53
RF
>Negative 54
RDMPIQAFLLY
>Negative 55
QNDKIHPFAQTQSLVYPFGPIPNSLPQNIPPLTQTPVVV
>Negative 56
GGLG
>Negative 57
QLFNPSTNPWH
>Negative 58
FYPELFR
>Negative 59
FYPE
>Negative 60
PR
